# Supplementary material for: Differential linear brain growth patterns in preterm neonates based on birth gestational age and steroid exposure: A retrospective chart review
Source: PLoS One. 2025 Jun 5;20(6):e0323454. doi: 10.1371/journal.pone.0323454 (PMC12140223; doi:10.1371/journal.pone.0323454)
Supplement: S1 File — (DOCX) [file pone.0323454.s001.docx]

Supplementary File 1: Measurements for brain linear metrics performed on cranial ultrasound at term equivalence age along with plane of measurement

|  | **Cerebral structure**  **(*Plane for measurement*)** | **Measurements** |
| --- | --- | --- |
| Cerebrum | | |
| 1 | Biparietal diameter  (*Coronal*) | The maximum diameter in the transverse plane at the level of the parietal eminences. |
| Cerebral white matter | | |
| 2 | Corpus-callosum length  (*Mid-sagittal*) | The distance between the genu and splenium of the corpus callosum |
| 3 | Corpus-callosum-fastigial distance  (*Mid-sagitta*l) | The length between the genu of the corpus callosum and the fastigium (roof of the fourth ventricle) |
| Deep grey matter | | |
| 4 | Basal ganglia width  *(Coronal)* | The maximum distance between the most lateral border of the basal ganglia and the midline. The lateral border of the basal ganglia was identified as being more echogenic than the adjacent white matter. |
| 5 | Caudate head width  *(Mid-coronal*) | Measured at right angles to a line that runs from its upper outer corner to the inner lower corner and the maximum distance between the ventricular wall and the lower margin of the caudate head that runs adjacent to the anterior limb of the internal capsule |
| Cerebellum | | |
| 6 | Cerebellar vermis height  (*Mid-sagittal*) | The distance between the upper and lower borders of the vermis |
| 7 | Transverse cerebellar diameter  (*Coronal*) | The maximum horizontal distance between the lateral borders of the cerebellar hemispheres |
| Brainstem | | |
| 8 | Pons anterior-posterior depth  (*Mid-sagittal plane*) | The distance between the anterior border of the pons at its maximal forward prominence and the posterior border at the level of the upper border of the 4th ventricle |
