# Supplementary figures and images for: Differential linear brain growth patterns in preterm neonates based on birth gestational age and steroid exposure: A retrospective chart review
Source: PLoS One. 2025 Jun 5;20(6):e0323454. doi: 10.1371/journal.pone.0323454 (PMC12140223; doi:10.1371/journal.pone.0323454)

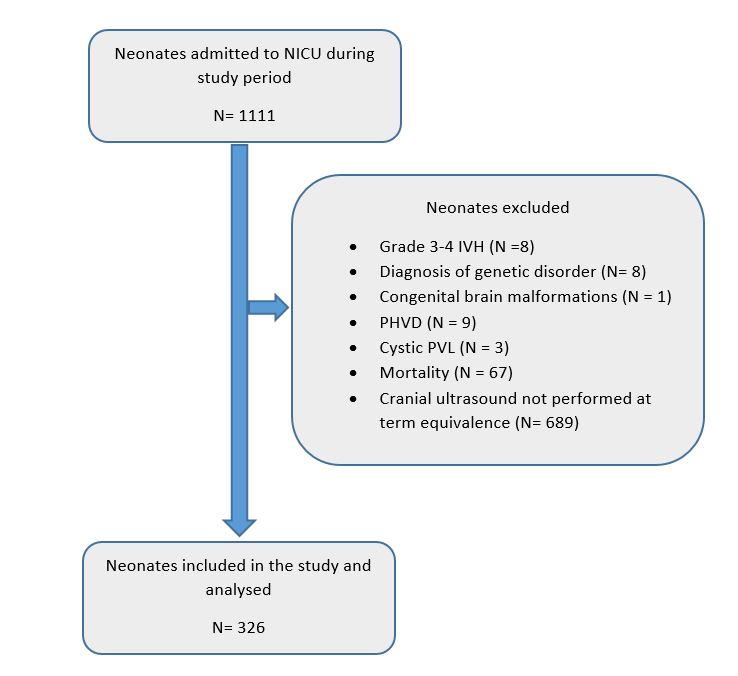

Supplement: S2 File — (JPG) [file pone.0323454.s002.JPG]

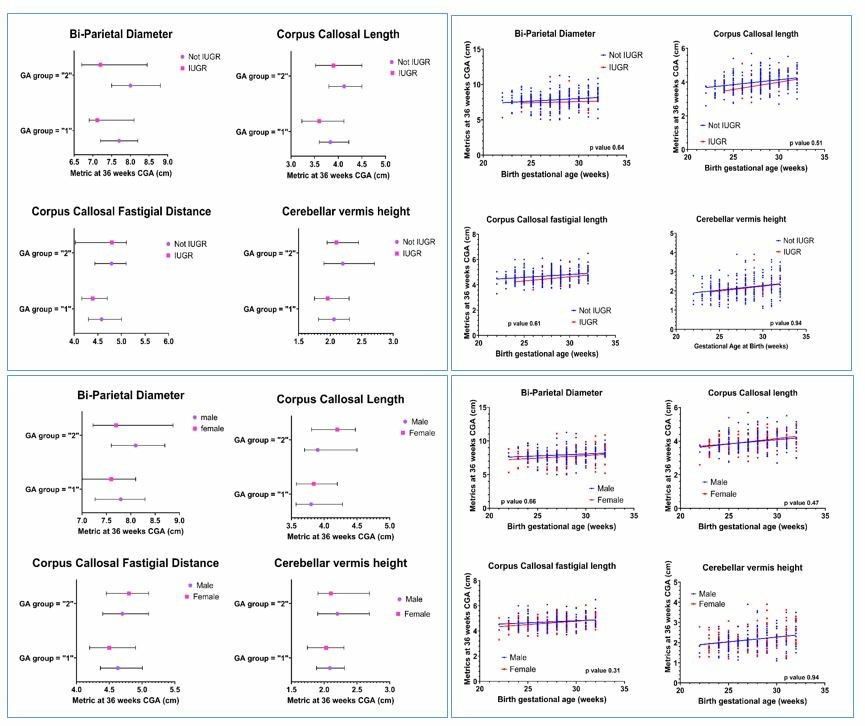

Supplement: S4 File — Bottom panel: (Left) Comparison of linear brain metrics between male and female EP (22–28 weeks GA) and VP (28+1–32 weeks GA) infants at term equivalence age; (Right) Simple linear regression of linear brain metrics of male and female infants. (JPG) [file pone.0323454.s004.jpg]
