# Supplementary material for: Differential linear brain growth patterns in preterm neonates based on birth gestational age and steroid exposure: A retrospective chart review
Source: PLoS One. 2025 Jun 5;20(6):e0323454. doi: 10.1371/journal.pone.0323454 (PMC12140223; doi:10.1371/journal.pone.0323454)
Supplement: S3 File — (DOCX) [file pone.0323454.s003.docx]

Supplementary File 3: Measurements for brain linear metrics on term equivalent cranial ultrasound that were not significantly different between Group 1 (22-28 weeks GA) and Group 2 (28^+1^-32 weeks GA)

| Brain metric  [Median,  (1^st^, 3^rd^ quartile)] | Group 1  [22-28 weeks GA] | Group 2  [28^+1^-32 weeks GA] | p-value |
| --- | --- | --- | --- |
| Basal-ganglia width | 1.90 (1.70, 2.10) cm | 1.92 (1.76, 2.14) cm | 0.20 |
| Caudate head width | 0.44 (0.35, 0.54) cm | 0.41 (0.33, 0.53) cm | 0.26 |
| Transverse cerebellar diameter | 4.80 (4.47, 5.20) cm | 4.90 (4.57, 5.40) cm | 0.19 |
| Pons anteroposterior depth | 0.71 (0.59, 0.88) cm | 0.72 (0.59, 0.85) cm | 0.85 |
