# Supplementary material for: Differential linear brain growth patterns in preterm neonates based on birth gestational age and steroid exposure: A retrospective chart review
Source: PLoS One. 2025 Jun 5;20(6):e0323454. doi: 10.1371/journal.pone.0323454 (PMC12140223; doi:10.1371/journal.pone.0323454)
Supplement: S5 File — (DOCX) [file pone.0323454.s005.docx]

**Supplementary File 5 (Table): Comparison of maternal and neonatal characteristics of neonates with steroid and without steroid exposure**

|  | **Any antenatal steroid exposure**  **N= 144** | **No antenatal steroid exposure**  **N= 182** | **P value** |
| --- | --- | --- | --- |
| Maternal age (years)^a^ | 31.53 ± 4.82 | 31.74 ± 5.90 | 0.725 |
| Maternal hypertension n(%) | 30 (20.8) | 30 (16.5) | 0.314 |
| Maternal diabetes n (%) | 19 (13.2) | 24 (13.2) | 0.998 |
| Gestation age (weeks) | 27.38 ± 2.55 | 27.62 ± 2.65 | 0.398 |
| Small for gestational age n (%) | 11 (7.6) | 17 (9.3) | 0.586 |
| Vaginal delivery n (%) | 47 (32.6) | 55 (30.2) | 0.640 |
| Delayed Cord Clamping n (%) | 82 (56.9) | 114 (62.6) | 0.297 |
| Male sex n (%) | 75 (52.1) | 94 (51.6) | 0.938 |
| Birth weight ^a^ | 1114.1 ± 428.97 | 1082.8 ± 411.09 | 0.504 |
| Apgar at 5 min ^b^ | 8 | 8 | 0.204 |
| Respiratory distress syndrome n (%) | 124 (86.1) | 134 (73.6) | **0.006** |
| Grade I-II IVH n (%) | 76 (52.8) | 107 (58.8) | 0.277 |
| Necrotizing Enterocolitis n (%) | 12 (8.3) | 12 (6.6) | 0.550 |
| Hemodynamically significant PDA n (%) | 49 (34.0) | 57 (31.3) | 0.604 |
| Culture proven sepsis n (%) | 41 (28.5) | 49 (26.9) | 0.756 |
| Bronchopulmonary Dysplasia n (%) | 99 (68.8) | 134 (73.6) | 0.333 |
| Median length of stay (days) ^b^ | 89.5 | 90 | 0.793 |
